# Nasal powders of thalidomide for local treatment of nose bleeding in persons affected by hereditary hemorrhagic telangiectasia

**DOI:** 10.1016/j.ijpharm.2016.07.002

**Published:** 2016-11-30

**Authors:** G. Colombo, F. Bortolotti, V. Chiapponi, F. Buttini, F. Sonvico, R. Invernizzi, F. Quaglia, C. Danesino, F. Pagella, P. Russo, R. Bettini, P. Colombo, A. Rossi

**Affiliations:** a Department of Life Sciences and Biotechnology, University of Ferrara, Via Fossato di Mortara 17/19, 44121 Ferrara, Italy; b Department of Pharmacy, University of Parma, Parco Area delle Scienze 27/A, 43124 Parma, Italy; c Department of Internal Medicine, University of Pavia, IRCCS Fondazione Policlinico San Matteo, Piazzale Golgi 19, 27100 Pavia, Italy; d Department of Molecular Medicine, General Biology and Medical Genetics Unit, University of Pavia, Via Forlanini 6, 27100 Pavia, Italy; e Department of Otorhinolaryngology, IRCCS Fondazione Policlinico San Matteo, Piazzale Golgi 19, 27100 Pavia, Italy; f Department of Pharmacy, University of Salerno, via Giovanni Paolo II 132, 84084 Fisciano, SA, Italy; g PlumeStars srl, Strada G. Inzani 1, 43125 Parma, Italy

**Keywords:** AVMs, arteriovenous malformations, HHT, hereditary hemorrhagic telangiectasia, SNES, simulated nasal electrolyte solution, THAL, thalidomide, Thalidomide, Telangiectasia, Nasal powders, Cyclodextrins, Mucoadhesion, Nasal devices

## Abstract

In this work nasal powder formulations of thalidomide were designed and studied to be used by persons affected by hereditary hemorrhagic telangiectasia as a complementary anti-epistaxis therapy, with the goal of sustaining the effect obtained with thalidomide oral treatment after its discontinuation for adverse effects. Three nasal powders were prepared using as carriers β-CD or its more hydrophilic derivatives such as hydropropyl-β-CD and sulphobutylether-β-CD and tested with respect to technological and biopharmaceutical features after emission with active and passive nasal powder devices. For all formulated powders, improved dissolution rate was found compared to that of the raw material, making thalidomide promptly available in the nasal environment at a concentration favouring an accumulation in the mucosa. The very limited transmucosal transport measured *in vitro* suggests a low likelihood of significant systemic absorption. The topical action on bleeding could benefit from the poor absorption and from the fact that about 2–3% of the thalidomide applied on the nasal mucosa was accumulated within the tissue, particularly with the β-CD nasal powder.

## Graphical abstract

**Figure:**
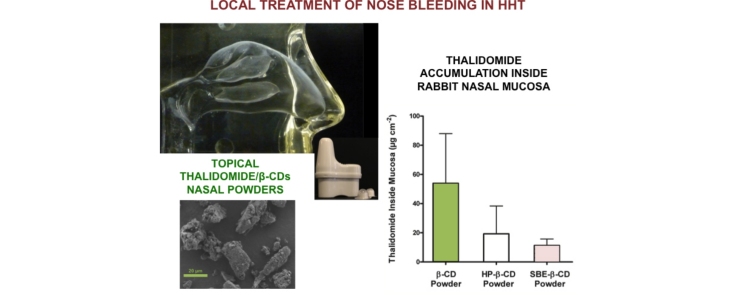

## 1 Introduction

Hereditary hemorrhagic telangiectasia (HHT) is a rare genetic disease characterized by vascular malformations with a prevalence of about 1–2 in 10,000 individuals (Govani and Shovlin, 2009). HHT is caused by mutations in genes encoding proteins mostly expressed in vascular endothelial cells. These proteins are involved in transforming the growth factor-beta superfamily signalling pathway. Clinical manifestations include arteriovenous malformations (AVMs), which can be classified as *small telangiectases* (regions of capillary dilation) and *large AVMs*, where arterioles and venules are directly connected with no capillaries in between. In particular, *small telangiectases* are typical of the nasal septum, oral mucosa and gastrointestinal tract, whereas *large AVMs* mainly occur in the lung, brain and liver. As these lesions are due not only to an expansion of the vascular lumen, but also to thinning of the vascular wall, they cause chronic nasal and/or gastrointestinal bleeding and severe anaemia. Indeed, recurrent epistaxis is the most common manifestation of nasal HHT and begins by the age of 10–20 years in many patients, increasing in severity with ageing. Epistaxis may be so severe as to require blood transfusions and oral iron supplementation (Guttmacher et al., 1995).

Treatments for epistaxis include surgical replacement of nasal epithelium with skin, laser ablation, topical application of anti-inflammatory drugs or manipulation of the coagulation and fibrinolytic pathways (Guttmacher et al., 1995). However, these options do not permanently resolve bleeding and are not well accepted by patients. Recent *in vivo* studies showed that oral administration of thalidomide reduced the frequency and duration of nose bleeds in HHT patients (Invernizzi et al., 2015, Lebrin et al., 2010). The anti-angiogenic and anti-hemorrhagic activities of thalidomide are due not only to a direct inhibition of endothelial cell proliferation and migration, but also to an increase in mural cell coverage, which stabilizes blood vessels and prevents bleeding. However, the observed anti-epistaxis effect of thalidomide is reversible and nose bleeding recurs after discontinuation of the oral treatment. Thus, systemic administration must be resumed, at the risk of causing adverse effects, such as reversible neuropathy, constipation and sedation (von Moos et al., 2003).

When HHT involves the nasal mucosa, the topical administration of thalidomide inside the nasal cavity for a maintenance treatment, complementing the systemic administration, is a therapeutic hypothesis. Thalidomide is practically insoluble in water and undergoes rapid and spontaneous hydrolysis in an aqueous environment (Goosen et al., 2002, Pereira et al., 2013). Therefore, thalidomide should be formulated as dry powder for insufflation, to increase physico-chemical and microbiological stability (Vidgren and Kublik, 1998) as well as drug concentration on and contact time with the nasal mucosa (Colombo et al., 2011, Jiang et al., 2010).

However, the performance of a powder formulation for nasal delivery will be highly dependent on drug dissolution rate in the liquid lining the nasal mucosa. Complexation with β-cyclodextrins (β-CDs) has been described as increasing thalidomide aqueous solubility (Kratz et al., 2012, Krenn et al., 1992). β-CD has proven to be an excellent solubility and absorption enhancer in nasal drug delivery (Challa et al., 2005, De Ascentiis et al., 1996, Merkus et al., 1999), being the most used in pharmaceutical applications since its cavity size is suitable for many drugs. Some β-CD derivatives used in topical drug formulations, such as hydroxypropyl-β-CD (HP-β-CD) and sulphobutylether-β-CD (SBE-β-CD), have higher water solubility and a better toxicological profile than β-CD (Badr-Eldin et al., 2008, de Araujo et al., 2008, Fukuda et al., 2008, Loftsson and Masson, 2001, Marttin et al., 1998).

Hence, in this work novel nasal powder formulations of thalidomide were studied to enable thalidomide administration in the nose and provide HHT patients treated by oral thalidomide with a complementary anti-epistaxis therapy. The ultimate goal is to sustain the effect obtained with the oral treatment after its interruption. Three powders were prepared using as carriers β-CD or the more hydrophilic HP-β-CD and SBE-β-CD, aiming to identify the best performing thalidomide nasal powder with respect to technological and biopharmaceutical features. In particular, thalidomide dissolution and transport across rabbit nasal mucosa and powder mucoadhesion were investigated. Then, as nasal drug administration requires a device for dose delivery, the powder formulations were combined with different devices to determine the plume emission and deposition profile in a model cast of the human nose. Studying the effect of thalidomide nasal administration in HHT in humans was out of our scope here and will be the subject of a future work.

## 2 Materials and methods

### 2.1 Materials

Thalidomide was purchased from Olon S.p.A. (Milan, Italy). Phenacetin was obtained from Carlo Erba (Milan, Italy). β-cyclodextrin and hydroxypropyl-β-cyclodextrin were supplied by Roquette (Lestrem, France). Sulphobutylether-β-cyclodextrin was kindly donated by CYDEX Pharmaceuticals (Lawrence, KS, USA). Lecithin (LIPOID S45) was supplied by Lipoid AG (Steinhausen, Switzerland). HPLC-grade acetonitrile was obtained from VWR International S.r.l. (Milan, Italy). All other reagents and solvents used were analytical grade.

### 2.2 Methods

#### 2.2.1 HPLC assay

Thalidomide quantification was performed by reverse phase high performance liquid chromatography (HPLC) using the two isocratic methods reported in Table 1. For drug quantification in powders and in phase solubility and dissolution rate samples, the method complied with the USP 34 Thalidomide Official Monograph. System suitability was assessed according to USP 34.

The method for determining thalidomide concentration in the transport and mucoadhesion experiments was developed in-house. This method was validated for repeatability (RSD 0.053% and 0.063%, for area and retention time values respectively; n = 6 injections) and limits of quantification and detection (LOQ 0.65 ng/ml; LOD 0.22 ng/ml; n = 3 injections).

#### 2.2.2 Phase solubility studies

Phase solubility studies of thalidomide in the presence of cyclodextrins were carried out in phosphate buffer solution 0.01 M pH 5.0. Excess amounts of thalidomide (about 50 mg) were added to 25 ml of buffer containing increasing concentrations of cyclodextrins. Dispersions were magnetically stirred for 5 h, at room temperature, then cleared through a 0.45 μm membrane filter and assayed for thalidomide concentration with the HPLC Method 1 (Table 1).

#### 2.2.3 Nasal powder manufacturing

Thalidomide-cyclodextrin powders were produced by a kneading technique. 2.5 g of thalidomide and 7.0 g of cyclodextrin raw materials were blended in a Turbula^®^ mixer (Type T2A; WAB, Muttenz, Switzerland). The blend was transferred to a mortar and kneaded with a solution of 0.5 g lecithin in 15 ml of ethanol. The wet mass in the mortar was oven dried at 40 °C up to complete solvent evaporation. The dried product was forced through a 0.4 mm mesh sieve. This procedure allowed for the preparation of three nasal powders containing thalidomide, lecithin and one of the three cyclodextrins (β-CD, HP-β-CD or SBE-β-CD) in the ratio of 25:5:70 (w/w).

#### 2.2.4 Powder physico-chemical characterization

Differential Scanning Calorimetry (DSC) measurements were performed on an indium calibrated DSC 821^e^ calorimeter (Mettler Toledo S.p.A., Milan, Italy). Accurately weighed samples (about 5 mg) were placed in aluminium pans, crimped and pierced. Samples were heated at a rate of 10 °C/min, from 30 to 300 °C, under a 100 ml/min flow of dry nitrogen. The resulting thermograms were analyzed with STARe Software to assess drug/excipient interactions.

Powder X-ray diffractometry (PXRD) patterns were recorded on a PW1050 diffractometer (Philips Analytical, Almelo, The Netherlands), equipped with a curved graphite crystal, using Cu Kα radiation. The scanning rate employed was 0.5° min^−1^ over a 2θ range of 2–50°.

Morphology of powders was analyzed by scanning electron microscopy (SEM) (Sigma HD, Carl Zeiss, Oberkochen, Germany) at EHT 1.00 kV. A double-sided adhesive tape was placed on an aluminium stub and a minimal amount of powder was dispersed by gently tapping on the edge of the stub. The samples were analyzed at different magnifications after 30 min of depressurization.

Particle size distribution of powders was measured using laser light diffraction apparatus (Spraytec, Malvern Instruments Ltd., Malvern, UK), by suspending powder aliquots in a 0.1% solution (w/v) of Span 85 in cyclohexane. The analysis was performed in a 100 ml cell under magnetic stirring, using a 300 mm lens.

Bulk and tapped volumes were determined with a 25 ml graduated cylinder with 0.5 ml graduation marks according to Ph. Eur., on powder samples weighing about 8–8.5 g. The bulk volume was read after a gentle manual tapping of the cylinder. The tapped volume was measured with a Tapped Density Tester (Erweka GmbH, Heusenstamm, Germany) after tapping sequences of 1250 taps. From bulk and tapped volumes, the Compressibility Index (or Carr Index, CI) was calculated according to the following formula:

$$CI=100(\nu_{0}-\nu_{f})/\nu_{0}$$

where *v_o_* is the bulk volume and *v_f_* is the tapped volume.

The static angle of repose was determined using the glass funnel technique according to the Ph. Eur. 8th Ed.

#### 2.2.5 Biopharmaceutical characterization

##### 2.2.5.1 *In vitro* dissolution rate

Thalidomide dissolution rate was measured using a USP Apparatus II dissolution tester (Erweka DT6R; Erweka GmbH, Heusenstamm, Germany). Samples of thalidomide-cyclodextrin powders, equivalent to about 5–6 mg thalidomide, were spread on the surface of the dissolution medium (900 ml of PBS 0.01 M pH 5.0) at 37 ± 0.5 °C and stirred at 100 rpm. At fixed time points, samples filtered through a 0.45 μm membrane filter were analyzed for thalidomide concentration by HPLC.

##### 2.2.5.2 *In vitro* transport across excised nasal mucosa

Thalidomide transport across excised rabbit nasal mucosa from the nasal powders was studied *in vitro* using vertical Franz-type diffusion cells (diffusion area: 0.58 cm^2^; Vetrotecnica, Padova, Italy). Rabbit nasal mucosa was extracted from rabbit heads supplied by a local slaughterhouse (Pola S.r.l., Finale Emilia, MO, Italy) on the day of the experiment and transported to the lab in a refrigerated box. The extraction procedure, completed within 2 h from the animal’s death, has been described elsewhere (Balducci et al., 2013). Franz cells were assembled with the tissue’s mucosal side facing the donor. The receptor compartment was filled with 5 ml of a solution composed of acetate buffer pH 5.0 and PEG 400 (50:50 v/v) to assure sink conditions. The assembled cell was left to equilibrate for 15 min at 37 °C before introducing the formulation.

A saturated solution of thalidomide in 50 mM sodium acetate buffer pH 5.0 was used as reference. One ml of this solution or 3 mg of nasal powder (corresponding to 750 μg of thalidomide) was introduced in the donor. With the powder, 100 μl of acetate buffer pH 5.0 was added to the donor for powder dissolution. The cell was protected from light during the experiment, which lasted 4 h (Bortolotti et al., 2009). This experiment duration was consistent with viability data in the literature for animal excised nasal tissue in the course of *in vitro* studies: 3–4 h for bovine nasal mucosa and up to 10 hours for isolated rabbit nasal mucosa with oxygenation (Bechgaard et al., 1992, Schmidt et al., 2000). The receptor solution was sampled only at the end of the experiment.

The residual thalidomide in the donor compartment was quantitatively recovered with aliquots of mobile phase for subsequent HPLC analysis (Method 2 in Table 1). Thalidomide accumulating within the tissue thickness was then recovered by comminuting the mucosa with a surgical blade and homogenizing in 5 ml of water with Ultra-Turrax^®^ IKA (T10 basic model, IKA^®^-Werke GmbH & Co. KG, Staufen, Germany) for 3 min. Two milliliters of methanol were then added and homogenization continued for a further 30 s to disrupt the cell membranes and dissolve the thalidomide. The homogenate was centrifuged and the supernatant injected as such. Thalidomide amounts recovered from receptor and donor compartments and the amount extracted from the mucosa allowed for calculating the mass balance (expressed as % of the thalidomide loaded in the donor). The number of replicates for these studies was between 6 and 10.

##### 2.2.5.3 *In vitro* mucoadhesion studies with rabbit nasal mucosa

The biological material for these experiments was supplied to and handled at the lab as described before. The nasal septum with the mucosa layering on the cartilage was extracted from the rabbit’s head and used as a surface for powder deposition. The septum with the lining mucosa was flat and rectangular (area 4.9 ± 0.8 cm^2^; mean ± S.D., n = 7).

The septum in a Petri dish was introduced into a glass desiccator at a relative humidity of 75–76% at room temperature (Wexler and Hasegawa, 1954). About 20 mg of thalidomide-cyclodextrin powder formulation were manually sprinkled on the whole available mucosa surface. The dish was covered and left in the desiccator for 20 min. Three Falcon™ 50 ml conical tubes were each filled with 15 ml of simulated nasal electrolyte solution (SNES) at room temperature. SNES contained 8.77 mg/ml sodium chloride, 2.98 mg/ml potassium chloride and 0.59 mg/ml calcium chloride dehydrate (Castile et al., 2013). 20 min after powder deposition, the nasal septum was pinched with tweezers on its shorter side. Holding it firmly, the septum was continuously submerged and withdrawn from the liquid in the tube for 1 min with a frequency of 1 s in and 1 s out. The procedure was repeated with the second and third tube for a total “washing time” of 3 min.

The suspensions in the three tubes were centrifuged to separate the solid (detached powder) from the supernatant (8000 rpm, 40 min). The sediments were treated with methanol to quantify thalidomide in the powder physically detached from the mucosa by the washing procedure, *i.e*., the non adhered thalidomide as solid. The supernatants were also analyzed by HPLC Method 2 (Table 1) to quantify the fraction of thalidomide solubilized by the SNES, *i.e*., the non adhered thalidomide as solution. Finally, the mucosa with the residual powder attached was treated with methanol to dissolve thalidomide for quantification (adhered thalidomide).

Mucoadhesion was expressed as percent of adhered thalidomide versus the total thalidomide recovered. For each powder formulation, at least 3 replicates were carried out.

#### 2.2.6 Powder and device combination (delivery and deposition in nasal cast)

The amount of nasal powder emitted upon device actuation was measured and pictures of the delivery sequence were taken (Buttini et al., 2012, Russo et al., 2004). Three devices were employed: one active single dose (MIAT, Milan, Italy), one active multi-dose (Teijin Ltd., Tokyo, Japan) and a passive bi-dose (Aptar, Louveciennes Cedex, France) device. MIAT and Aptar devices are designed for delivering a dose of powder pre-metered in a size 3 capsule and in a bi-dose blister reservoir respectively. Both were loaded with approximately 20 mg of powder, corresponding to a 5-mg thalidomide dose. The Teijin device has an internal reservoir for the powder bulk and a metering chamber. MIAT and Teijin devices have a squeezable rubber bulb manually actuated by an operator to generate the airflow. Since the Aptar device is breath-actuated, 15 L/min airflow was drawn through it for 2 s using a vacuum pump to extract the powder from the reservoir. Quantitative delivery of the powder from the insufflator was determined by weighing the insufflator before and after each actuation. Sequences of images of the powder plume during the actuation were recorded using a video camera (Panasonic HDC-TM 700, Panasonic Corp., Kadoma, Japan).

Plume deposition in a human nasal model was carried out using a silicon cast (Teijin Pharma Ltd. Tokyo, Japan) (Bettini et al., 1999). Insufflation with the same three devices was performed in one nostril of the assembled nasal cast. The nasal cavities were then separated and pictures were taken.

#### 2.2.7 Powder stability study

The stability of thalidomide-cyclodextrin powders was studied in the short term, namely for 3 months of storage at 25 °C/60% RH and for 1 month at 40 °C/75% RH. About 20 mg of each formulation, corresponding to a 5-mg thalidomide dose, were stored in size 3 HPMC capsules and tested at fixed time points for thalidomide content, *in vitro* dissolution rate and powder insufflation using the single-dose MIAT device.

#### 2.2.8 Statistical analysis

Statistical analysis of data was performed by applying an unpaired two-tailed Student’s *t*-test. Significance was accepted at p values < 0.05.

## 3 Results and discussion

Nasal powders of thalidomide have to induce a local anti-angiogenic action on the microvasculature of the nasal mucosa compromised by HHT. To exert a local effect or to have drug absorption, the formulation must enable drug dissolution in the small volume of liquid lining the mucosa. On site powder dissolution leads to local drug saturation concentration. Thalidomide is very slightly soluble in water and the raw material does not wet easily. Thus, it was decided to introduce a cyclodextrin in the formulation as a solubility enhancer (Kale et al., 2008, Kratz et al., 2012, Krenn et al., 1992).

**Fig. 1:**
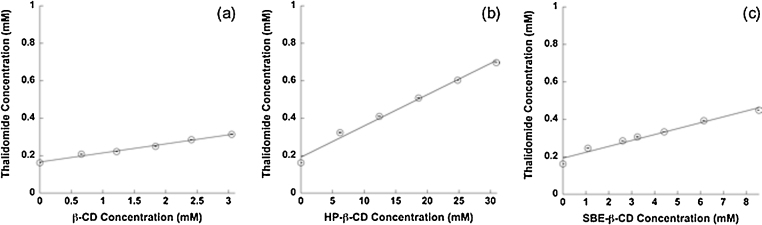
Phase solubility diagrams of thalidomide in presence of (a) β-CD, (b) HP-β-CD and (c) SBE-β-CD (mean values ± S.D.; n = 3). Linear fitting: (a) y = 0.16626 + 0.04830 x (R^2^ = 0.99), (b) y = 0.19209 + 0.01669 x (R^2^ = 0.99) and (c) y = 0.19313 + 0.03153 x (R^2^ = 0.97).

In order to establish the optimal thalidomide to β-CD, HP-β-CD or SBE-β-CD ratio in the nasal product, phase solubility studies were carried out in phosphate buffer pH 5.0 at 25 °C. This buffer was used as solvent because thalidomide is more stable at acidic pH. All three β-CDs increased thalidomide solubility, which was about 42 μg/ml in the absence of cyclodextrin. The phase solubility diagrams show that thalidomide solubility increased linearly as a function of cyclodextrin concentration (Fig. 1). The relationship between cyclodextrin and thalidomide concentrations led to an A_L_-type diagram, which is typical of 1:1 complexes. Calculated stability constants were 313 M^−1^, 105 M^−1^ and 201 M^−1^ for β-CD, HP-β-CD and SBE-β-CD respectively. The complexation efficiency of the three cyclodextrins, whose inverse value expresses the number of CD molecules in solution that form a 1:1 complex with thalidomide, was 0.05 for β-CD, 0.017 for HP-β-CD and 0.032 for SBE-β-CD (Loftsson et al., 2005).

### 3.1 Manufacturing and physico-chemical characterization of thalidomide/cyclodextrin powders

β-CD, HP-β-CD and SBE-β-CD were used for the formulation of the nasal powder because they all enhanced thalidomide solubility in aqueous medium to different extents. The three nasal powders were produced by kneading and contained 25% thalidomide (w/w) and 70% cyclodextrin (w/w) with the objective of having 5 mg of drug in 20 mg of powder to insufflate. This meant that the molar ratio between thalidomide and the CD was 1:0.64, 1:0.53 and 1:0.51 respectively for β-CD, HP-β-CD and SBE-β-CD. The formulation also contained 5% (w/w) lecithin, which was added during kneading as ethanol solution to improve the wettability of thalidomide.

**Fig. 2:**
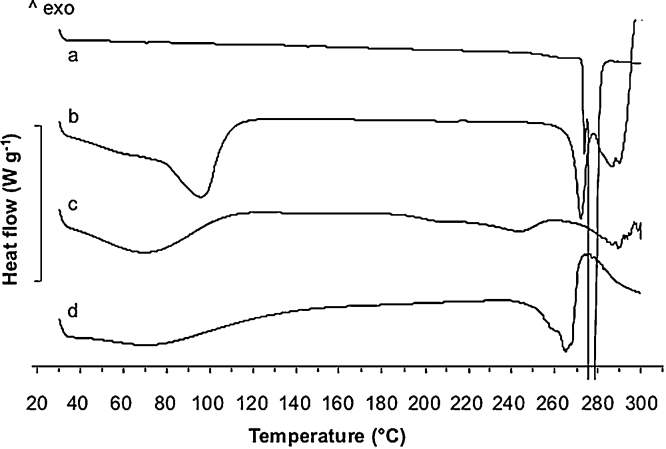
DSC thermograms of (a) thalidomide raw material, (b) β-CD nasal powder, (c) HP-β-CD nasal powder and (d) SBE-β-CD nasal powder.

In order to verify whether a complex was formed during the kneading process, the powders were studied by differential scanning calorimetry in comparison with crystalline thalidomide (THAL) raw material (Fig. 2). The THAL raw material thermogram showed a sharp endothermic peak at 276 °C corresponding to the drug melting point. The thermograms of the thalidomide/CD powders showed a broad endothermic band around 100 °C, corresponding to the release of water from the cyclodextrin cavity. The melting peak of thalidomide was still present, although much smaller and shifted to lower temperatures, likely due to an interaction of the two compounds in the mixture. In fact, the enthalpy of fusion for the pure drug was −158 J g^−1^, whereas it was −44.32 J g^−1^, −21.92 J g^−1^ and −133.94 J g^−1^ respectively for THAL/β-CD, THAL/HP-β-CD and THAL/SBE-β-CD. This last value should not be considered as fully reliable, being affected by the degradation peak of SBE-β-cyclodextrin that superposed to the THAL melting peak.

Additional evidence of an interaction occurring between thalidomide and cyclodextrins was obtained by PXRD analysis (Supplementary Material). Thalidomide raw material peaks were sharp and intense, confirming its crystalline nature. In contrast, thalidomide kneaded with the cyclodextrins showed less intense peaks.

**Fig. 3:**
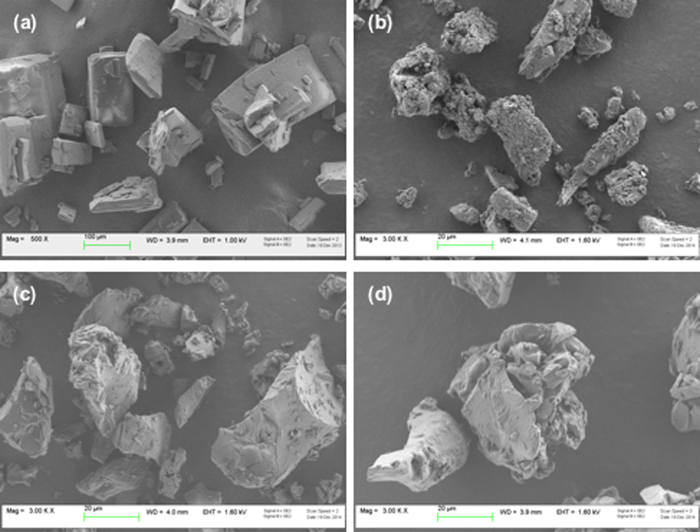
SEM images of (a) thalidomide raw material, (b) β-CD nasal powder, (c) HP-β-CD nasal powder and (d) SBE-β-CD nasal powder.

Powder particle size, packing and flow are key properties in view of nasal dosage form manufacturing, delivered dose uniformity, aerodynamic behaviour during insufflation, dissolution rate and interaction with the wet mucosa. Particle size analysis by laser diffraction showed a mean volume diameter (D_v0.5_) between 13 and 22 μm for all powders (Table 2). This size was also confirmed by SEM analysis (Fig. 3). The particle shape of kneaded products appears as agglomerates with sharp edges, resulting from the sieve comminution after drying. Nevertheless, this non spherical morphology did not negatively affect powder flow and packing. Bulk density and repose angle values of these nasal powders allowed for classifying the formulations containing β-CD and HP-β-CD as free-flowing according to the Pharmacopoeia (Table 2). The SBE-β-CD nasal powder had inferior flow properties and looser packing.

### 3.2 Nasal powder stability

For thalidomide-cyclodextrin powders stored in size 3 HPMC capsules at 25 °C/60% RH for 3 months and at 40 °C/75% RH for 1 month, β-CD powder and HP-β-CD powder remained stable in all conditions with respect to thalidomide content. Similarly, SBE-β-CD powder did not show a significant decrease in thalidomide content, despite its colour turning to pale yellow after storage at 40 °C. Stability was also confirmed with respect to *in vitro* dissolution rate and powder delivery.

### 3.3 Biopharmaceutical characterization

#### 3.3.1 *In vitro* thalidomide nasal powder dissolution rate

Dissolution rate of thalidomide nasal powders was measured *in vitro* in an aqueous medium at pH 5.0 over 2 h (Goosen et al., 2002, Pereira et al., 2013, Schumacher et al., 1965). These experiments were carried out with the purpose of comparing the dissolution behaviour of the nasal powders in sink conditions for the quality assessment of solid preparations. Thalidomide raw material, *i.e*., the solid material before the formulation process, was used as reference.

**Fig. 4:**
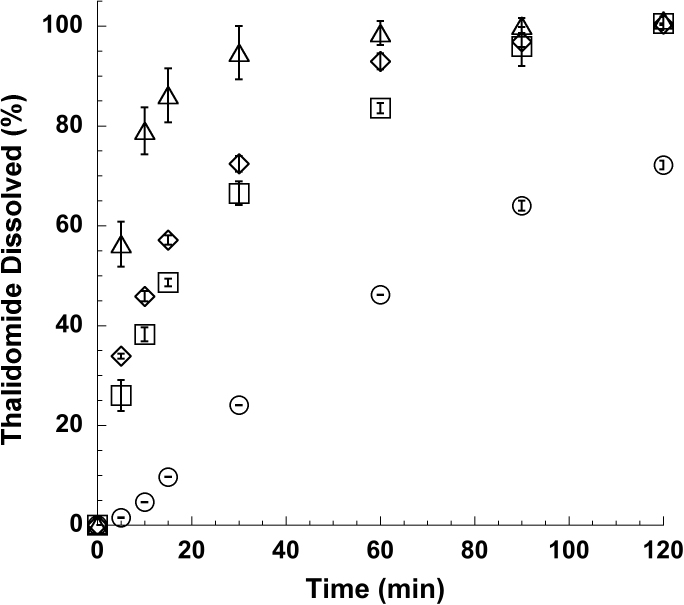
Dissolution profiles of (○) thalidomide raw material, (□) β-CD nasal powder, (△) HP-β-CD nasal powder and (◊) SBE-β-CD nasal powder (mean ± S.D.; n = 3).

In these conditions only 46.0 ± 0.1% of thalidomide raw material dissolved in 1 h, reaching 72.0 ± 0.9% at the end of the second hour (Fig. 4). It was observed that during the first minute drug powder floated on the dissolution medium surface, owing to its difficult wettability. In contrast, kneaded powders containing cyclodextrins together with lecithin dissolved more rapidly, reaching 84 ± 1%, 99 ± 2% and 93 ± 2% respectively of thalidomide in solution after 60 min. for the powder containing β-CD, HP-β-CD and SBE-β-CD. This improvement in drug dissolution rate had two main explanations. Firstly, solid state analysis of the powders showed that thalidomide and cyclodextrins partially interacted. This interaction at the molecular level increased the drug solubility in aqueous environment (as demonstrated in the phase solubility studies). The second positive contribution to thalidomide dissolution rate came from lecithin, owing to its surface-active properties. The positive effect of lecithin on wettability increased the powder surface interacting with the solvent.

#### 3.3.2 *In vitro* thalidomide transport across rabbit nasal mucosa

**Fig. 5:**
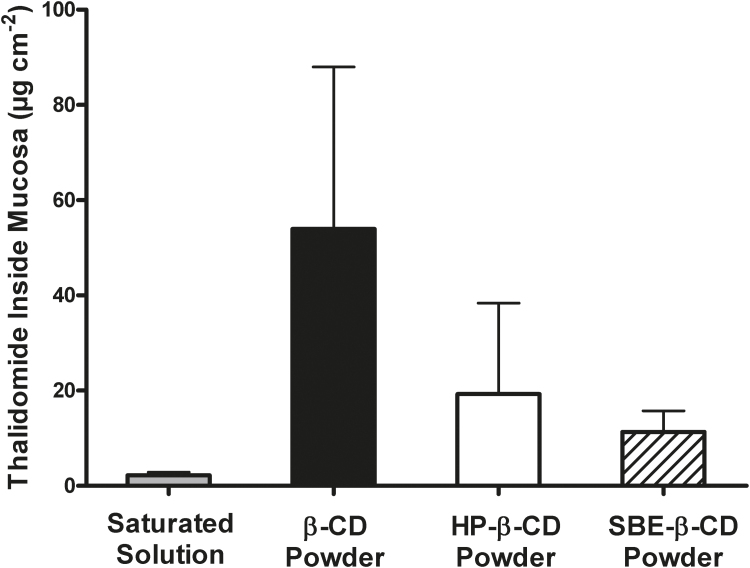
Amounts of thalidomide extracted from the mucosa at the end of the 4-h diffusion experiment (thalidomide loaded at time zero: 65 μg for the saturated solution, 750 μg for the powders; mean ± S.D., n = 6–10).

*In vitro* THAL transport across excised rabbit nasal mucosa in a 4-h experiment was negligible from all powders, as well as from the thalidomide saturated solution. Having observed that transmucosal diffusion was very low, if not absent, the THAL concentration in the receptor compartment was measured only at the end of the experiment. THAL concentration was above LOQ only in the experiments with the thalidomide saturated solution and the nasal powder containing β-CD (0.09 ± 0.10 μg/5 ml and 0.13 ± 0.09 μg/5 ml respectively). Independently of the formulation, more than 80% of the loaded thalidomide remained in the donor. The thalidomide present in the mucosa at the end of the permeation experiment was extracted from the tissue sample used as barrier. The amounts recovered are shown in the bar graph of Fig. 5.

Any possible drug degradation occurring during the permeation was assessed by determining the thalidomide mass balance after all transport experiments (Table 3). The mass balance for the powder formulations was higher than 90%, indicating that the experimental variability and drug stability were under control. The mass balance below 90% for the saturated solution has to take into account that the amount of thalidomide loaded in the donor was much lower than with the powders (65 vs 750 μg respectively).

In summary, the transport experiments showed that thalidomide was poorly diffused across the nasal epithelium, remaining to a certain extent in the tissue. The negligible transport across the mucosa is a pivotal result with respect to topical application, because it reduces the likelihood of thalidomide being significantly absorbed from the nasal cavity into the systemic circulation. In full awareness that these *in vitro* data cannot substitute the bioavailability study following nasal administration, in any case they indicate that *in vivo* systemic absorption may not be clinically relevant. In fact, these data were collected after 4 h of contact between formulation and mucosa, whereas residence time inside the nasal cavity, even for a solid formulation, may not be as long. Indeed, it must be borne in mind that the damaged microvasculature of the nasal mucosa in HHT is the target of the local application of thalidomide. Thus, thalidomide accumulation within the tissue is favourable to its local action.

Comparing the nasal powder formulations, the β-CD powder led to the greatest THAL accumulation within the tissue. β-cyclodextrin is known as an absorption enhancer in nasal drug delivery (Marttin et al., 1998). The mechanism underlying this action has been related to the interaction with the nasal epithelial membranes and transient opening of tight junctions. Several studies indicate different enhancing properties of β-cyclodextrin and derivatives depending on the substituting moiety. In general, methylated derivatives appeared more effective compared to hydroxypropyl-β-cyclodextrin with respect to nasal absorption of drugs (Merkus et al., 1999). The higher absorption enhancing effect of β-cyclodextrin compared to the other derivatives was also seen from the traces of thalidomide detected in the receptor compartment.

#### 3.3.3 Mucoadhesion

Thalidomide interaction with cyclodextrins improved powder mucoadhesion compared to the drug raw material as powder. In this work the mucoadhesion was measured by a methodology consisting in washing, in controlled conditions with a simulated nasal fluid (SNES), a sample of nasal mucosa on which the nasal powder had been previously deposited. The results are expressed as mean values ± standard deviation of replicates carried out by two operators. The obtained variability of the individual data is consistent with a reproducible procedure.

**Fig. 6:**
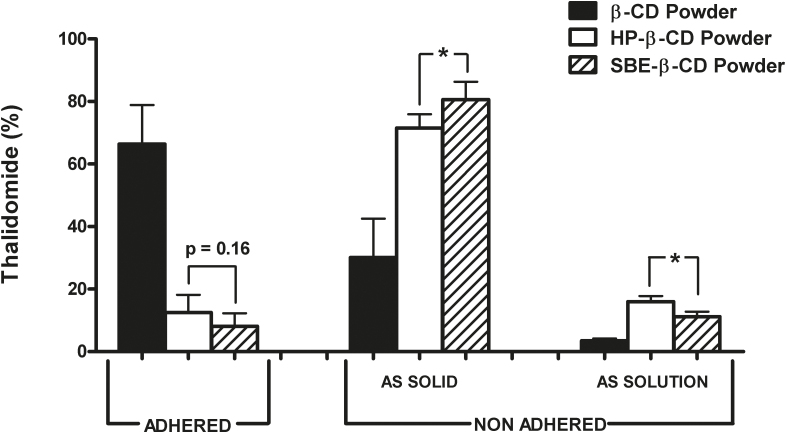
Percentage of thalidomide retained on (ADHERED) and removed from the mucosa surface (NON ADHERED) after 3 min washing in 45 ml of simulated nasal fluid. The non adhered amounts were recovered as dispersed solid or in solution in the washing fluid; mean ± S.D., n = 3–4. *p < 0.05 according to unpaired two-tailed Student’s *t*-test.

For the three formulations, the average percentage of THAL retained on the mucosa surface after the washing procedure with the simulated nasal fluid (ADHERED) and the drug amounts not retained and recovered in the removed solid or dissolved in the fluid (NON ADHERED) are shown in Fig. 6. To reveal a possible time dependency of mucoadhesion, the product was removed from the mucosa by washing three times at 1 min intervals. It was found that the lower the thalidomide dissolution rate in water, the greater the mucoadhesion. Indeed, 50% of the β-cyclodextrin powder was still retained on the mucosa surface after the washing. This was the same powder enabling the highest thalidomide accumulation within the mucosal tissue (see Fig. 5). As the more hydrophilic HP-β-CD and SBE-β-CD increased wettability and dissolution rate in aqueous media, the corresponding nasal powders more easily lost the contact with the mucosa and were removed. The respective adhered amounts were not significantly different (p = 0.16). The solid material physically detached from the mucosa remained dispersed or dissolved in the washing liquid phase. In fact, between 4% (β-CD powder) and 16% (HP-β-CD powder) of removed thalidomide was found in solution. Thus, dissolution by the washing fluid was mostly relevant for the most soluble HP-β-CD powder (p < 0.05 vs SBE-β-CD powder).

From a kinetic point of view, most powder detachment and dissolution occurred during the first minute of washing, in particular for the HP-β-CD and SBE-β-CD powders (80 ± 5% and 82 ± 8% respectively; p = 0.23). Then, between 3% and 6% of thalidomide was washed away during the second and third minute, without significant differences between the above two powders. In contrast, a time-dependency of mucoadhesion was not found for the β-CD powder, whose total non adhered fraction was mainly recovered as dispersed solid in SNES (Supplementary material).

### 3.4 Powder and insufflation device combination

A nasal drug product is a typical combination product, comprising formulation and device for its delivery. The choice of a proper device is driven by the therapy requirements and target deposition site, as well as the characteristics of the product to be administered. For the local action desired in HHT, thalidomide deposition should cover the maximum mucosal surface for the longest possible time. Moreover, the device should effectively deliver the powder, while limiting its dispersion in the environment. In this regard, in the case of thalidomide a passive device may be preferable.

In order to propose a nasal device tailored to the investigated formulation, powder delivery and deposition were tested with three commercially available devices with different insufflation mechanisms, namely an active single-dose (MIAT), an active multi-dose (Teijin) and a disposable passive bi-dose (Aptar) one. The three devices operated at markedly different insufflation airflows, *i.e*., 4, 0.6 and 15 L/min respectively. We wanted to investigate whether and in what way the actuation mode and airflow affected powder dose delivery and deposition site inside the nose cast.

Table 4 presents the powder amounts delivered upon actuation of each of the considered devices. The reported values are the mean of ten shots. Capsules or blisters used in MIAT and Aptar contained 20 mg of powder, corresponding to a thalidomide dose of 5 mg. The delivered dose for the multi-dose device (Teijin) was the average of ten consecutive metered actuations.

Powder delivery depended on both formulation technological properties and device airflow during insufflation. With MIAT and Teijin devices the delivered dose was in line with the powder repose angle values (HP-β-CD > β-CD > SBE-β-CD; see Table 2). The differences in delivered amount coming from the powder flow characteristics were levelled down with the Aptar device. This was reasonably due to the higher operating airflow. The powder formulated with HP-β-CD was the only one quantitatively emitted without significant differences among the devices. The values were within the limits (± 15% of the label claim) of the guideline on the pharmaceutical quality of inhalation and nasal products (EMA, 2006).

Picture sequences of emitted plumes show that powder delivery from the devices was completed in less than one second (Supplementary Material). The plume development with Aptar was difficult to visualize owing to the experimental need to confine it in an air drawing tube connected with the nose adapter. The combined effect of formulation and device variables was reflected in the plume shape. The more flowable powders containing β-CD and HP-β-CD led to closely packed plumes. In contrast, the more cohesive SBE-β-CD powder generated less dense clouds, confirming a less efficient powder delivery for this formulation.

Taking into account the disease, the target deposition site and desired intranasal distribution of the drug, a final study was carried out generating the powder plume with the three insufflators directly into a silicone human nasal cast. The cast allowed for visualizing differences among the distribution of the powders in a human nasal cavity. Only the HP-β-CD powder was considered for this study because the delivered amount was the same for all devices (see photographs of deposition patterns in the Supplementary Material). Each device gave rise to a different degree of coverage of the various regions of the nasal cavity. During the experiment, we qualitatively observed that the Aptar and Teijin devices deposited the powder more homogeneously in the vestibular, pre-turbinate and turbinate regions, whereas in the MIAT device larger particles were deposited in the turbinate region.

## 4 Conclusions

The nasal powder formulation of thalidomide with lecithin and β-cyclodextrin or its derivatives hydroxypropyl-β-cyclodextrin or sulphobutylether-β-cyclodextrin gave rise to stable products with suitable technological properties for nasal insufflation. From a biopharmaceutical point of view, the improved dissolution rate compared to that of the raw material made thalidomide promptly available in the aqueous nasal environment at a concentration giving rise to an accumulation in the mucosa, with limited transmucosal transport. This *in vitro* observation suggests a low likelihood of significant systemic absorption. The topical action on bleeding could benefit from the poor absorption and from the fact that about 2–3% of the thalidomide applied on the mucosa *in vitro* was accumulated within the tissue, particularly with the β-CD nasal powder. Furthermore, residence time in the nasal cavity may be prolonged by the mucoadhesive action provided by this nasal powder. Considering the nasal cavity coverage, deposition of β-CD nasal powder was predicted as more efficient when using a passive device, *i.e*., a device delivering the powder only when the patient inhales through it. An additional advantage of this actuation mode could be the limited powder dispersion in the environment around the patient. This is relevant with a formulation containing thalidomide. Thus, in nose telangiectasia, nasal powders could enable topical thalidomide treatment as a complement to systemic oral administration for maintenance therapy, thus contributing to a safer re-positioning of an effective active drug.

## Author disclosure statement

The authors declare no conflict of interest.

## Figures and Tables

**Table 1:** Conditions of the HPLC methods used.

Conditions	Method 1 (USP34)	Method 2 (in-house)
Apparatus	Chromatopac LC-10AS pump, ESA temperature-controlled autosampler and SPD-10 UV detector (Shimadzu, Kyoto, Japan)	Agilent 1100 series with auto-sampler and UV–vis detector (Agilent, Santa Clara, CA, USA)
Stationary phase	C18 Synergi Hydro 4 μm column (150 × 3.9 mm)^a^	C18 Luna 3 μm column (150 × 4.6 mm)^a^
Mobile phase	Water: acetonitrile: H_3_PO_4_ 85:15:0.1 (v/v)	50 mM NaH_2_PO_4_: Acetonitrile 60:40 (v/v); pH 3
Internal standard (IS)	Phenacetin	–
Flow rate (ml/min)	1	0.6
Detection wavelength (nm)	237	220
Temperature (°C)	25 °C	Ambient
Injection volume (μl)	50	40
Thalidomide retention time (min)	18 (24 for IS)	4.8

a Supplied by Phenomenex (Torrance, CA, USA).

**Table 2:** Particle size and powder flow of thalidomide/cyclodextrin powders (n = 3).

Nasal Powder	D_v0.1_ (μm)	D_v0.5_ (μm)	D_v0.9_ (μm)	Bulk Density (g/ml)	Tapped Density (g/ml)	Compressibility Index	Angle of repose
β-CD	3.1 ± 0.1	19.2 ± 0.5	58.5 ± 2.9	0.55 ± 0.01	0.70 ± 0.01	20.0	28.5 ± 1.2
HP-β-CD	3.2 ± 0.2	18.1 ± 2.1	61.6 ± 5.7	0.60 ± 0.01	0.80 ± 0.01	20.0	21.8 ± 2.6
SBE-β-CD	3.5 ± 0.3	21.2 ± 3.5	61.1 ± 10.3	0.48 ± 0.01	0.60 ± 0.01	23.3	34.6 ± 0.7

**Table 3:** Mass balance from *in vitro* transport experiments in Franz cells (mean ± S.D.; number of replicates in parentheses).

Formulation	Mass balance (%)
Saturated solution (10)	83 ± 7
β-CD (6)	96 ± 6
HP-β-CD (6)	91 ± 2
SBE-β-CD (7)	96 ± 2

**Table 4:** Mean delivered powder amount (mg) from Aptar, MIAT and Teijin devices (mean ± S.D., *n* = 10).

Powder	Aptar	MIAT	Teijin
β-CD	20.4 ± 1.2	19.0 ± 0.7	18.5 ± 0.6
HP-β-CD	21.0 ± 1.7	21.0 ± 0.7	21.7 ± 1.2
SBE-β-CD	20.4 ± 1.2	17.6 ± 1.9	13.9 ± 0.8
